# An Investigation into the Effects of Correlated Color Temperature and Illuminance of Urban Motor Vehicle Road Lighting on Driver Alertness

**DOI:** 10.3390/s24154927

**Published:** 2024-07-30

**Authors:** Quan Chen, Zelei Pan, Jinchun Wu, Chengqi Xue

**Affiliations:** School of Mechanical Engineering, Southeast University, Suyuan Avenue 79, Nanjing 211189, China; genie85@163.com (Q.C.); 13814132940@163.com (Z.P.); wjcseu@seu.edu.cn (J.W.)

**Keywords:** urban motor vehicle road lighting, non-visual effects, driver alertness, EEG, reaction time

## Abstract

Current international optical science research focuses on the non-visual effects of lighting on human cognition, mood, and biological rhythms to enhance overall well-being. Nocturnal roadway lighting, in particular, has a substantial impact on drivers’ physiological and psychological states, influencing behavior and safety. This study investigates the non-visual effects of correlated color temperature (CCT: 3000K vs. 4000K vs. 5000K) and illuminance levels (20 lx vs. 30 lx) of urban motor vehicle road lighting on driver alertness during various driving tasks. Conducted between 19:00 and 20:30, the experiments utilized a human-vehicle-light simulation platform. EEG (β waves), reaction time, and subjective evaluations using the Karolinska Sleepiness Scale (KSS) were measured. The results indicated that the interaction between CCT and illuminance, as well as between CCT and task type, significantly influenced driver alertness. However, no significant effect of CCT and illuminance on reaction time was observed. The findings suggest that higher illuminance (30 lx) combined with medium CCT (4000K) effectively reduces reaction time. This investigation enriches related research, provides valuable reference for future studies, and enhances understanding of the mechanisms of lighting’s influence on driver alertness. Moreover, the findings have significant implications for optimizing the design of urban road lighting.

## 1. Introduction

Since the discovery of intrinsically photoreceptive retinal ganglion cells (ipRGCs) in mammalian eyes [1,2], there has been an escalating focus on investigating the non-visual effects of lighting. The research reveals that, upon the perception of light signals by ipRGCs, transmission occurs to the suprachiasmatic nucleus (SCN), orchestrating the regulation of the individual’s circadian rhythm system and hormone secretion. Concurrently, through the associative relationships between SCN and various regions of the cerebral cortex (such as raphe, amygdala, lateral hypothalamus, etc.), the light signals prompt a series of neural projection responses, thereby adjusting aspects of human physiology and psychology, including alertness, emotions, cognitive functions, and behavior [3,4,5]. Alertness is considered a cognitive state characterized by heightened preparedness and receptiveness to incoming sensory stimuli, resulting in swifter and more precise responses to external cues [6]. Maintaining a high level of alertness is essential for drivers, as it enables the prompt detection of subtle environmental changes, thereby significantly reducing the risk of accidents and allowing for swift and accurate reactions to potential hazards, thus enhancing overall road safety [7,8,9]. Within this context, this study aims to provide insights into the influence of the association between the non-visual effects of urban motor vehicle road lighting and driver alertness, an area where related research is relatively limited. 

The impact of light on cognition is determined by several key factors, including optical parameters such as illuminance and spectral composition (CCT), temporal characteristics like the timing and duration of exposure, and the nature of the cognitive task [5]. Previous research has consistently demonstrated that short-wavelength blue light (approximately 460–480 nm) exerts a profound influence on non-visual effects related to alertness [4,5,10]. The majority of research findings suggest that high CCT, associated with short-wave blue light, or high illuminance can enhance human alertness [11,12,13,14]. However, there are also studies that yield completely opposite conclusions [15]. Additionally, some research indicates that altering illuminance or CCT may not have a significant impact on human alertness [16,17]. Consequently, consensus on the impact of light on alertness in current research remains elusive. Furthermore, beyond the inherent properties of light, there is growing evidence suggesting that the nature of cognitive tasks may also modulate the sensitivity of non-visual effects produced by light on individual cognition [17,18,19].

Despite significant advancements in comprehending the non-visual impacts of road lighting on driver alertness within the research field, limitations persist due to various factors. Firstly, current research predominantly focuses on the non-visual effects of tunnel lighting on drivers, with limited investigation into outdoor road segments such as urban motor vehicle roads [20,21,22]. This is because the lighting conditions and physical environment within tunnels are more easily simulated in laboratory settings, facilitating experimental studies while ensuring participant safety. However, urban motor vehicle roads present more complex conditions and a higher likelihood of unexpected incidents, necessitating higher driver alertness. Therefore, studying the non-visual effects of nighttime urban motor vehicle road lighting is crucial to enhance our understanding and improve road safety. Secondly, current studies often utilize melatonin levels and reaction time as primary indicators for assessing driver alertness [23]. While these measures provide valuable insights, they have notable limitations. Melatonin levels, though indicative of circadian rhythms, may not directly correlate with moment-to-moment fluctuations in alertness during driving tasks. Reaction time, while a useful metric, offers a limited scope, as it does not capture the underlying neural processes influencing driver alertness. To address these shortcomings, incorporating physiological indicators such as electroencephalography (EEG) can significantly enhance the reliability and precision of evaluations. EEG provides real-time data on brain activity, offering a more comprehensive understanding of cognitive states [24]. This integration allows for a nuanced analysis of how different lighting conditions impact neural mechanisms underlying alertness, leading to more robust and actionable research findings. Thirdly, relevant studies are carried out mostly during the daytime [21,23]. Nevertheless, the rate of traffic collisions at night is significantly higher than during the daytime [25] and the non-visual impacts of light on the human circadian rhythm system exhibit distinct variations throughout different time intervals within a day [26]. Therefore, it is necessary to carry out a special study on nighttime hours. Notably, few previous studies have explored the interactive effects of illuminance and CCT, both of which are pivotal determinants in characterizing the non-visual effects of light [21]. Therefore, this research endeavors to shed light on the combined influences of illuminance and CCT of road lighting on driver alertness during nighttime driving activities. This study constitutes one component of the research project focusing on the comprehensive evaluation criteria and methodologies for urban road lighting. The ultimate objective is to provide scientific assessment methods and design recommendations for the holistic illumination of urban roads, with the aim of rendering urban road lighting more pleasant, environmentally friendly, efficient, and artistic in the future. The findings of this research provide valuable recommendations for assessing the utility of illuminance and CCT of urban motor vehicle road lighting to enhance driver alertness and task performance, contributing to the optimization of design solutions for road lighting.

## 2. Methodology

### 2.1. Design

The experiment was conducted in a 3 × 2 × 3 within-subjects repeated measures design; experimental variables and specific parameter settings are shown in Table 1. To mitigate the potential influence of the sequence of lighting conditions on the experimental outcomes, we implemented two key strategies. Firstly, a washout period of one day was imposed between each experimental session, ensuring that participants were exposed to only one lighting environment per session. This arrangement necessitated each participant to complete six distinct experimental sessions, with a day of rest interposed between each session. Secondly, to balance the order effects, a Latin square design was employed to systematically vary the sequence of the different CCT and illuminance combinations across participants. This methodology guarantees a balanced exposure sequence, thereby minimizing the risk of systematic biases influencing our results. This laboratory investigation was performed from September to December 2022.

### 2.2. Participants

A total of 18 healthy volunteers (M ± SD = 34.6 ± 0.73, 15 males, 3 females) holding valid driving licenses were recruited from Nanjing Lighting Construction and Operation Group Co., Ltd for this research. On average, participants had 7.2 ± 0.82 years of driving experience. Sample size determination utilized G*power 3.1.9 with a significance level (α) set at 0.05 and a medium effect size (f) of 0.25. Analysis indicated a minimum sample size requirement of 10 individuals to achieve a statistical power level of 80%. A comprehensive interview assessment confirmed the absence of mood disorders and chronic ailments among all participants. All participants had either normal vision or vision corrected to normal, assessed through standard vision tests, including the Ishihara color test. In the week preceding the experiment, participants were instructed to maintain their regular daily routines, minimize prolonged exposure to unique lighting (e.g., monochromatic light), ensure adequate sleep (approximately 8 h), and abstain from stimulant beverages such as coffee and strong tea. These precautions were taken to ensure that sleep quality, light exposure, and caffeine consumption did not influence the accuracy of EEG measurements by affecting participants’ baseline neural activity. All participants signed an informed consent form and received monetary compensation of 270 RMB for their participation after the experiment. 

### 2.3. CCT and Illuminance Setting Basis

#### 2.3.1. Measurement of Actual Road Lighting Parameters

**Figure 1:**
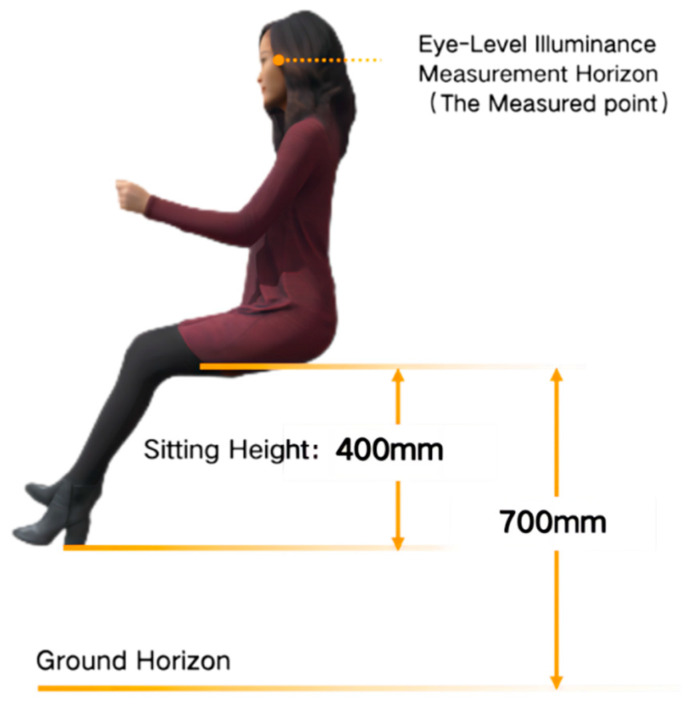
Diagram of eye-level illuminance measurement points.

To faithfully replicate the lighting environment of urban motor vehicle road within a laboratory setting and control deviations between simulated and actual lighting conditions, this research employs measurements of static and dynamic illuminance values (including road surface illuminance and driver’s eye-level illuminance) obtained from actual road lighting. These measurements serve as the basis for configuring optical parameters in simulating the lighting environment for experiments. Specifically, eye-level illuminance refers to the illuminance measured at the horizontal position where the driver’s eyes are aligned when seated and looking forward (as illustrated in Figure 1). This parameter authentically reflects the illuminance values of light projected into the interior of the human eye, making it a critical efficacy-based visual metric for evaluating non-visual effects. Therefore, by assessing the relationship between the actual road surface illuminance provided by streetlights and the driver’s eye-level illuminance inside the vehicle, it is possible to scientifically control the simulation of the laboratory lighting environment, achieving a higher degree of fidelity.

**Figure 2:**
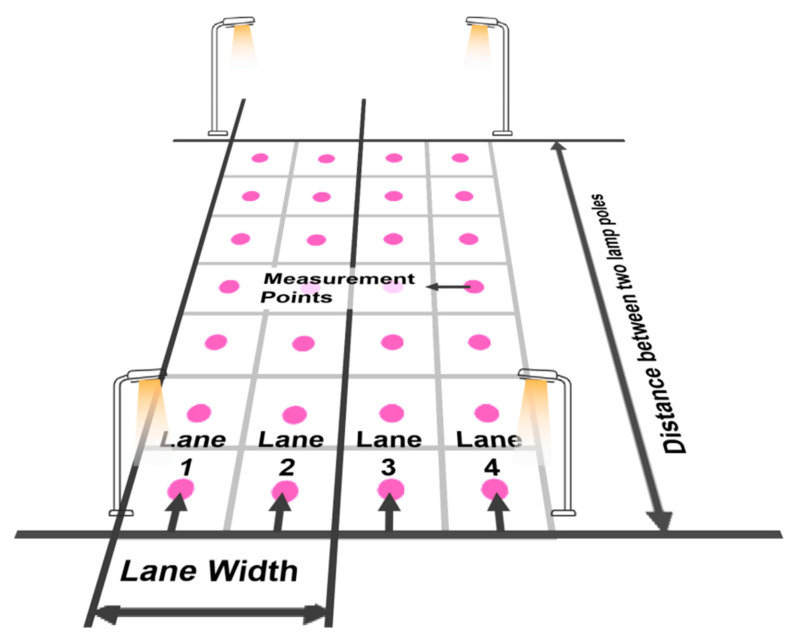
Schematic diagram of the measurement method for road surface illuminance.

This study adopted the measurement method of traditional road photometry indicators (central point method). The sampling points were selected on two road sections, namely Southeast University Road in Jiangning District and Shuanglong Avenue in Nanjing, both of which are four-lane two-way roads. The measurement methods are detailed in Figure 2.

The measurement of illuminance utilized an illuminance meter (Victor-VC1010D), with a sampling rate of 1 sample per second, capable of continuously collecting 50 data points. The experimenters conducted measurements during the evening from 19:00 to 20:30, capturing eye-level illuminance values of the drivers inside the vehicle (driver’s eye height from the ground is approximately 70 cm) and actual maintained average illuminance of road surface.

Specifically, the two roads under investigation are situated in the periphery of Southeast University, a location relatively secluded from the bustling city center. Consequently, during the period from 19:00 to 20:30, traffic volumes are comparatively low, rendering the impact of vehicle headlights on our measurements negligible. To further mitigate any potential interference, we ensured that static illuminance measurements were conducted in areas devoid of passing vehicles, thereby minimizing the disturbance from headlights. During the measurement process, if dynamic illuminance readings were recorded during vehicle passage (which typically exhibit elevated values), they were systematically identified and excluded from our analysis. This methodology ensures the accuracy of our research findings.

Static illuminance refers to the average illuminance measured at specified reference points on the road surface while the vehicle is stationary. Dynamic illuminance denotes the continuously measured average illuminance obtained at a frequency of 1 time/s as the vehicle travels at a speed of 60 km/h. Ultimately, A total of 428 data points were obtained for eye-level illuminance (400 dynamic illuminance, 28 static illuminance, with detailed statistical results provided in Table 2). 

#### 2.3.2. Illuminance Setting Basis

In accordance with optical principles, the relationship between the illuminance of an illuminated object surface and the distance from the light source is typically expressed through a proportional relationship, as indicated by Equation (1) [27]. Specifically, as the distance between the illuminated object and the light source increases, the illuminance on the surface of the illuminated object decreases. Hence, it can be deduced that under the condition of controlling other physical influencing variables (e.g., object occlusion, material of the illuminated object, etc. are consistent), there also exists a simple ratio relationship between the average illuminance of the road surface and the illuminance of the eye level (which is defined as *K* in this study; see Equation (2)), which serves as a crucial reference metric for guiding and examining simulated outdoor road lighting environments within the laboratory, ensuring that illuminance settings closely align with real-world scenarios to the greatest extent feasible. It is essential to note that, given the practical context, the majority of the time, drivers operate in dynamic driving conditions. Consequently, in this study, dynamic eye-level illuminance is selected for the computation of *K*. Here, *E* represents illuminance (lx), *I* represents the light intensity, defined as the luminous flux per solid angle, and *D* is the distance from the observation point to the light source (meters). *E_(eye-level)_* denotes the illuminance at the eye level of the driver inside the vehicle, while *E_(road surface)_* represents the average illuminance of the road surface.

(1) $$E=\frac{I}{D^{2}}$$

(2) $$K=\frac{E_{\left(eye-level\right)}}{E_{\left(road\;surface\right)}}$$

In this study, the parameter settings for illuminance conform to the Chinese urban road lighting design standard (China Academy of Building Research, 2016), with illuminance set at two levels: 20 lx (the minimum average illuminance standard for urban motor vehicle main roads) and 30 lx (the maximum average illuminance standard for urban motor vehicle main roads).

Substituting the measurement results from Table 2 into Equation (2), the calculated value of *K* is approximately 0.112 (*K* = 3.2/28.5). Therefore, when the average illuminance of the road surface is 30 lx, substituting this into Equation (2) (*K* ≈ 0.112), the corresponding eye-level illuminance value can be calculated to be approximately 3.4 lx. Similarly, when the average illuminance of the road surface is 20 lx, the calculated corresponding eye-level illuminance value is approximately 2.2 lx (values measured during the actual experiment were: 2.3 lx (eye level)—20 lx (road surface); 3.45 lx (eye level)—30 lx (road surface), compliance with experimental requirements).

#### 2.3.3. CCT Setting Basis

The current design specifications for the CCT of urban motor vehicle roads in China (China Academy of Building Research, 2016) recommend a standard guideline that the CCT should not exceed 5000K. However, these specifications provide limited details. Therefore, in this study, the configuration of the CCT is, on one hand, based on the actual CCT distribution in urban motor vehicle road lighting (CCT range of urban motor vehicles in Nanjing: 2700K~6000K, data sourced from Nanjing Lighting Construction and Operation Group Co. Database). This provides a basis for delineating the approximate range of CCT settings (2500K~6500K). On the other hand, reference is made to relevant studies [28,29,30,31] and our research team’s related work [32,33], providing a basis for selecting which CCT parameters to focus on (2500K, 3000K, 3500K, 4000K, 4500K, 5000K, 5500K, 6000K, 6500K) and for setting the difference between the different CCT levels (usually set to 500K or 1000K). Consequently, in the experiments conducted in this study, three CCT levels were selected (3000K, 4000K, 5000K).

### 2.4. Setting

#### 2.4.1. Physical Environment Settings

A darkroom (10 m × 20 m × 3.8 m) was constructed to replicate urban motor vehicle road lighting conditions. The windows were covered with blackout stickers to prevent outside distracting light from entering. Moreover, the walls and ceiling were enveloped in light-absorbing flocking fabric of photographic-grade quality, chosen for its capacity to minimize internal light reflections. In pursuit of simulating the diffuse reflective characteristics akin to asphalt concrete pavement, asphalt sheets were used to pave the ground.

The interior of the darkroom is equipped with a comprehensive driving simulation system, which encompasses a driving simulator (seat height: 70 cm) and a simulated driving scenario design program, underpinned by SACNeR Studio 2022.2 software. The integration of these components affords drivers an immersive simulated nighttime driving experience (the display offers a wide horizontal field of view of approximately 135°). 

**Figure 3:**
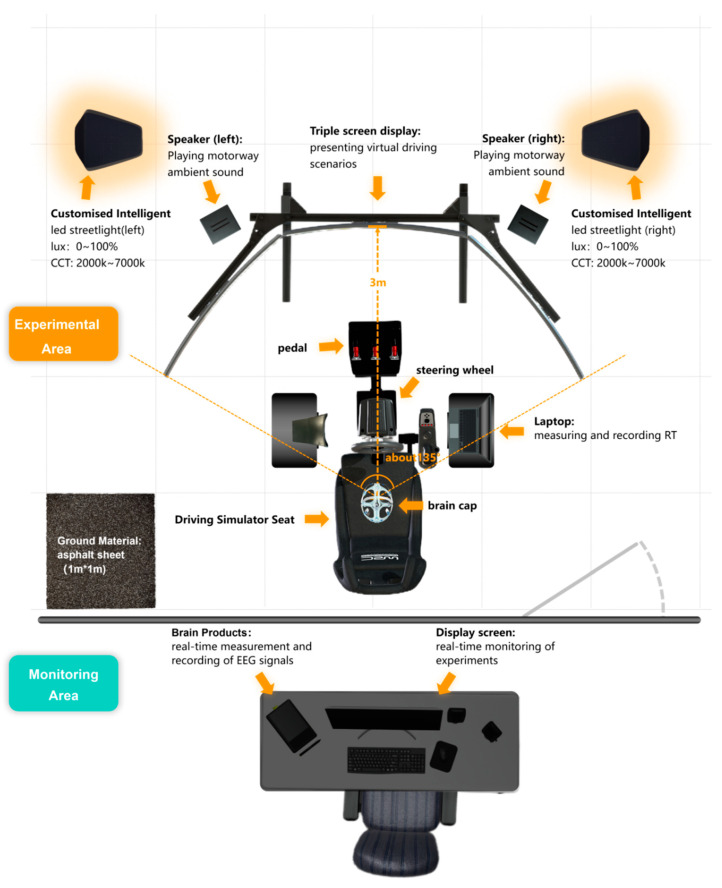
Top view of the layout of the simulation platform.

**Figure 4:**
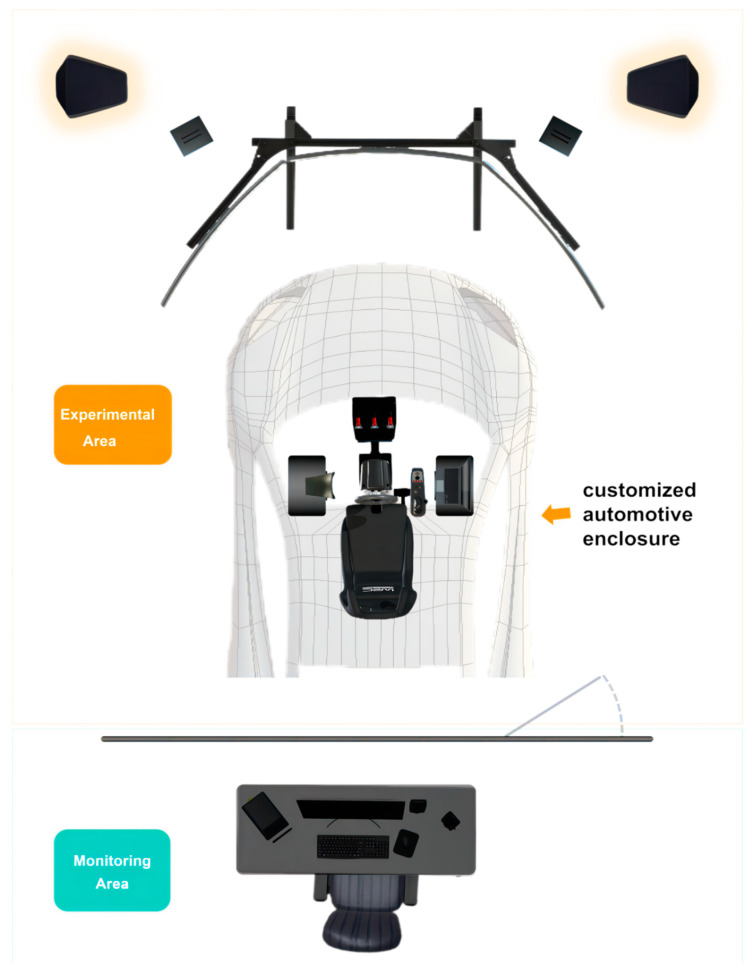
Schematic diagram of the solution for the control of illumination in the laboratory.

**Figure 5:**
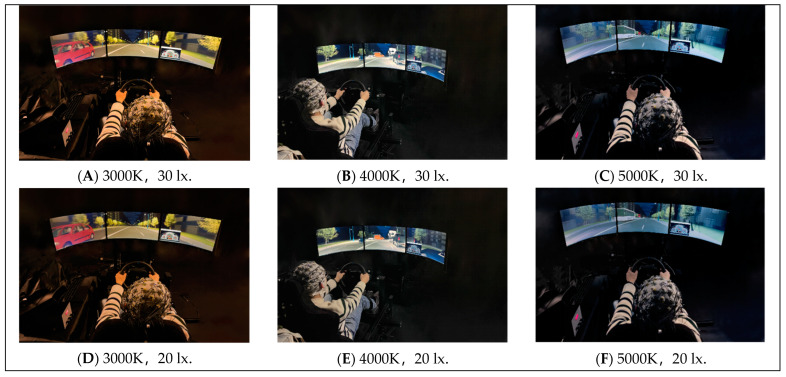
Photographs of scenes from experiments with different lighting schemes.

In addition, the laboratory is equipped with Brain Products (Munich, Germany) to comprehensively measure the physiological and psychological characteristic changes in the participants throughout the experiment. The related equipment and its functional description are shown in Figure 3. It should be noted that, in order to meet the illuminance requirements for simulating experimental conditions, a customized automotive enclosure was additionally installed (Figure 4). Figure 5 depicts the participants performing the experimental tasks in different simulated lighting during the experiment.

#### 2.4.2. Light Environment Settings

**Figure 6:**
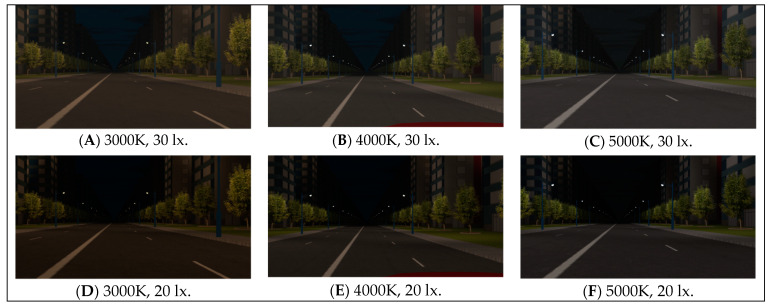
3D modeling of virtual driving scenes with different light environment scenarios.

**Figure 7:**
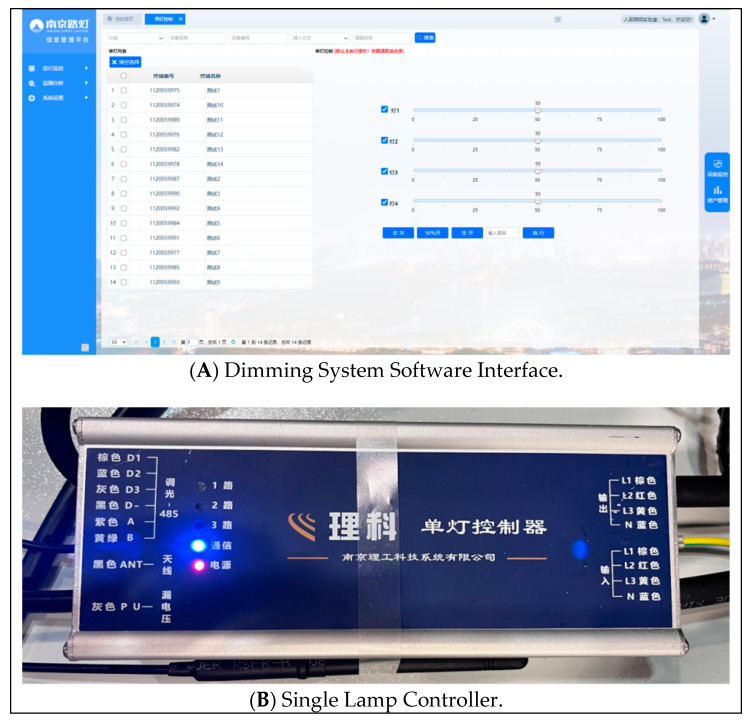
Software and hardware for intelligent dimming module sets.

The light environment in the laboratory consists of two main components: the primary light source provided by the LED streetlight dimming system and the virtual 3D nighttime driving environment (see Figure 6) presented on the screen. The LED streetlight dimming system comprises a control module, fixtures, lampshades, and mounting brackets. Utilizing the dimming control module (as shown in Figure 7), operators can remotely adjust the CCT (2000K~7000K) and illuminance (0%~100%) of the fixtures in real time.

In this research, the virtual light source model with RGB values and illuminance (see Table 3) equivalent to the actual luminaires was created using 3D Max to ensure that the light environment in the virtual driving scene provided an equivalent visual experience to the subjects as the actual fixtures in the darkroom, in order to control for the influence of visual effects on the results of the experiment.

In addition to the direct effects of correlated color temperature (CCT) and illuminance on drivers’ alertness and reaction time, it is essential to consider the potential influence of light pulsation and the power supply method of the light source on EEG brain activity. Previous studies [34] have demonstrated that the CCT and pulsation frequency of light emitted from fluorescent lamps and LEDs can significantly impact bioelectrical brain activity compared to traditional tungsten filament bulbs. Specifically, certain power supply methods and CCT values may introduce additional EEG emissions, notably in the 21–22 Hz range corresponding to Beta waves. However, it is important to note that not all supply methods and CCT values exhibit such effects. In our study, we employed a PWM power supply method, which we believe minimized potential interferences with EEG recordings while ensuring stable light flux emission.

**Figure 8:**
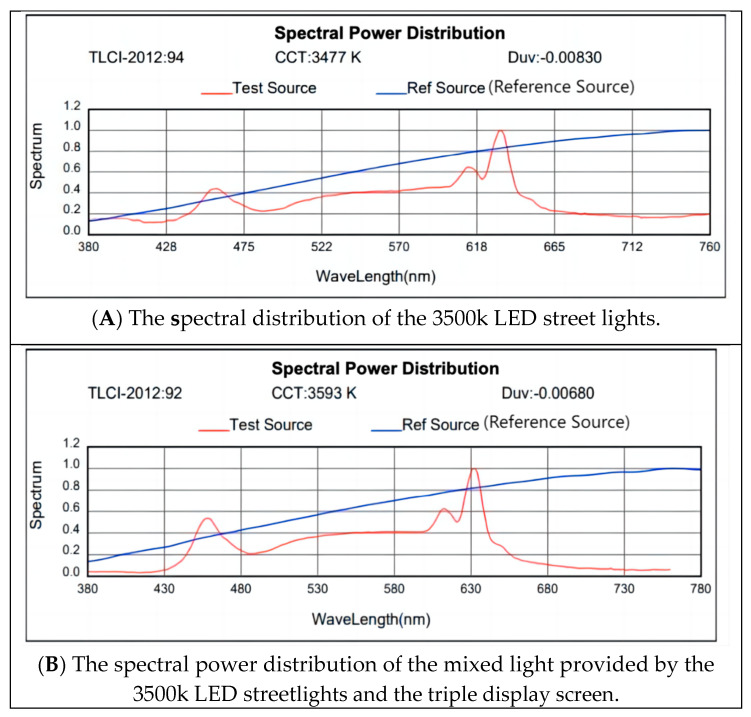
Difference in spectral distribution between single light and mixed light.

To mitigate and control the impact of the display’s lighting, the screen illumination was adjusted to a level of 10% (screen illumination: 9.5 lx, eye level illumination: approx. 0.67 lx). The photometric measurements indicate that the spectral distribution characteristics of the combined light from the screen and the luminaires (referring to LED streetlights) closely approximate those of the light from the luminaires alone, within the range of approximately 440 nm to 630 nm. Hence, it can be inferred that both the non-visual effects induced by mixed light (from the screen and luminaires) and single light (from luminaires) exhibit similar characteristics, thereby validating the scientific rigor of the experimental setup. It is imperative to explicitly state that the SPD test point was positioned immediately adjacent to the subject’s eye, horizontally aligned with it, and situated 3.0 m away from the center of the screen and 0.9 m above the ground. This positioning ensures that the spectral distribution measured by the SPD accurately reflects the light entering the subject’s eye. Figure 8 illustrates the difference between mixed light and single light (using a 3500K as an example).

### 2.5. Experimental Tasks and Procedure

#### 2.5.1. Experimental Tasks

**Figure 9:**
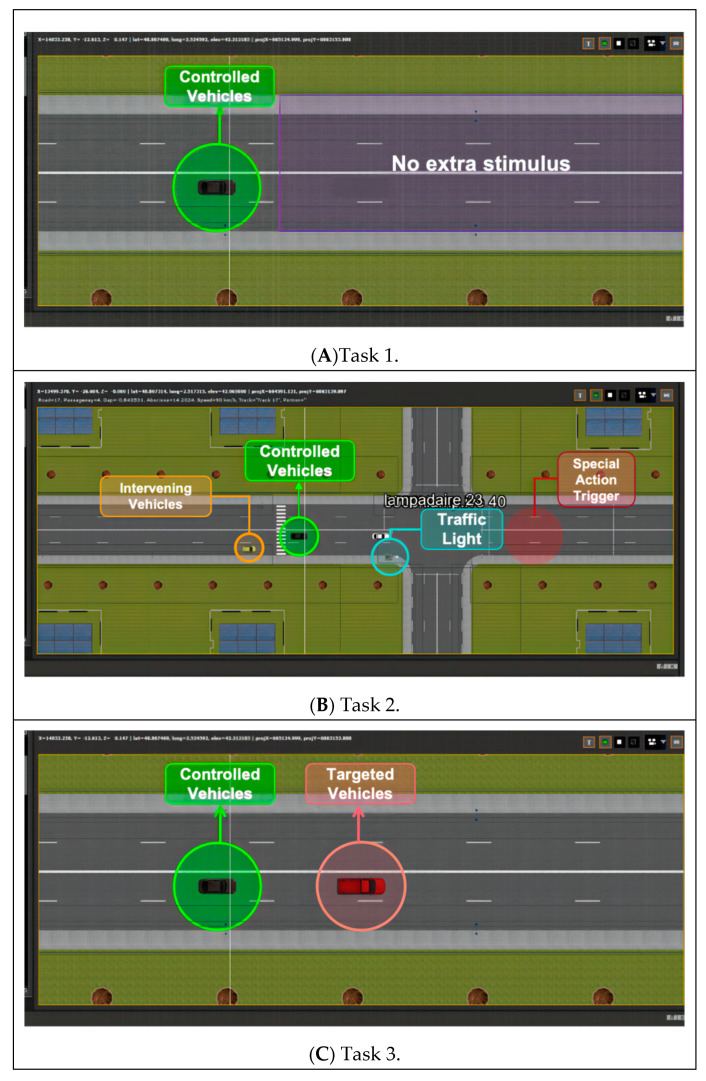
Task setup instructions (screenshot of SCANeR Studio interface).

In this study, three specific daily driving scenarios were selected as fundamental templates to design the simulated tasks (Figure 9). The aim was to replicate the real driving experience as closely as possible. During the simulated driving experiment, participants were instructed to drive along a straight road. It should be noted that with SCANeR Studio, the surrounding traffic density was set to ρ = 0.6, which represents a simulated value of the traffic flow on the urban motor vehicle road in the evening from 19:00 to 20:30.

(1) **Task 1 (baseline—monotonous driving):** Task 1 phase (baseline) was devoid of any unusual stimuli (e.g., traffic lights, obstacles, etc.) to simulate a monotonous driving environment. Participants were instructed to sustain a naturally relaxed state, manage the virtual vehicle at 60 km/h, adhere to traffic regulations, and avoid changing lanes.
(2) **Task 2 (waiting for red lights and traffic jams):** Task 2 simulated a driving scenario involving frequent waits at red lights and sporadic instances of random traffic congestion. Throughout this phase, the vehicle’s speed limit remained consistent at 60 km/h.
(3) **Task 3 (follow-up task and auditory PVT task):** Task 3 employed a dual-task experimental paradigm (primary task: follow-up task, secondary task: auditory psychomotor vigilance task (aPVT)) [35] to amplify perceptual load and diminish the likelihood of slower or inaccurate responses due to reduced attention. In this phase, participants were tasked with controlling the simulated vehicle to trail a specified target vehicle (a red pickup truck) while maintaining a self-assessed safe distance. If the target vehicle changed lanes, participants were required to steer the vehicle accordingly without overtaking or tailgating, and no specific speed limit was imposed. Furthermore, concurrently managing the following task, participants were prompted to react to randomized acoustic stimuli (a simulated ringtone resembling an incoming call) by swiftly pressing a designated key (designated as the space bar).

#### 2.5.2. Procedure

**Figure 10:**
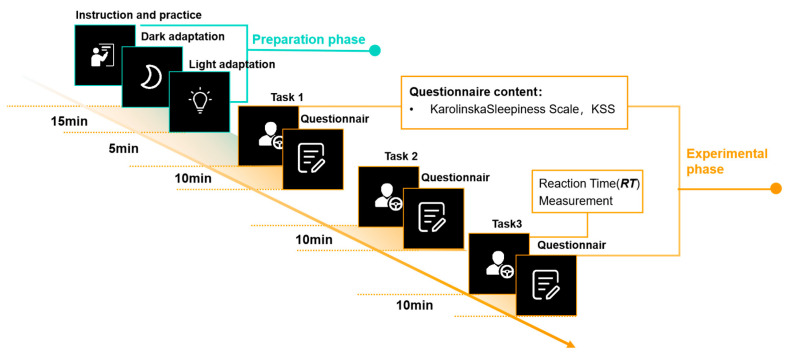
The procedure of the experiment.

One week before the experiment, participants received operational training on the driving simulator to ensure proficient handling of the equipment. Prior to the formal experiment, investigators provided participants with a detailed explanation of the experimental procedures and task execution requirements. Following the practice phase, participants wore completely occlusive eyeshades, closed their eyes, and underwent a 15-min dark adaptation in a dark environment to eliminate the potential impact of prior lighting conditions on the experimental results. After the dark adaptation period, participants underwent a 5-min period of light adaptation to acclimate to the current experimental lighting environment. Upon the completion of the light adaptation phase, the formal experiment commenced, during which participants’ EEG signals were collected and recorded in real time. Each participant underwent the experimental tasks in the following sequential order: Task 1, Task 2, and Task 3. Additionally, after completing each task, participants were required to fill out the KSS questionnaire. Figure 10 illustrates the procedure of the experiment.

### 2.6. Measures

#### 2.6.1. EEG Indicators (β)

Numerous studies have demonstrated a strong correlation between electroencephalogram (EEG) readings and assessments of alertness tasks [11,36,37,38]. The Beta (*β*) frequency band, ranging from approximately 14 Hz to 30 Hz, represents the EEG activity that is typically associated with states of nervousness, agitation, or arousal [37,39]. *β* power, measured in microvolts squared per hertz (μV²/Hz), serves as a highly sensitive indicator for detecting changes in driver alertness during driving sessions [39,40]. Specifically, in this research, the average power values of the *β* band, collected from electrode locations (P3, P4, PO3, PO4, O1, Oz, O2) [41,42,43] that follow the international 10–20 system, were employed as an assessment index to evaluate the influence of various lighting configurations on driving alertness. This assessment index was used to quantify the effect of different lighting setups on driver alertness.

#### 2.6.2. Task Performance (RT)

**Figure 11:**
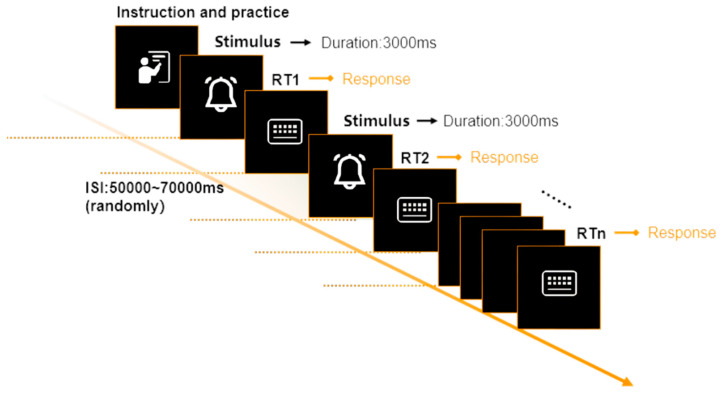
The procedure of the aPVT.

The Auditory Psychomotor Vigilance Task (aPVT), a prevalent psychological experimental paradigm, primarily aims to assess an individual’s proficiency in maintaining sustained attention and executing rapid responses [44,45]. During this task, participants are instructed to respond promptly and precisely to auditory stimuli, such as sound signals, either by pressing a button or executing a designated bodily movement. This paradigm has been extensively utilized in investigating cognitive and behavioral performances, particularly under conditions of sleep deprivation, fatigue, and vigilance decrement. To facilitate the development of the aPVT’s procedure, the software E-prime 3.0 was employed. During Task 3, the average reaction time (*RT*) of participants in response to auditory stimuli was collected to assess the sustained attention and cognitive control of drivers. Each auditory stimulus lasted 3000 ms, a duration determined as optimal through pre-experiment measurements to ensure drivers could respond within this time, maintaining data validity. The interval between stimuli, referred to as the inter-stimulus interval (ISI), was randomly set between 50,000 and 70,000 ms to prevent participants from predicting stimulus occurrence, thereby ensuring accurate measurements of reaction to unexpected events. Reaction time was recorded from stimulus onset to the participant pressing a button. All participants had normal hearing, verified using a standard audiometric test. Stimuli were presented at a standardized loudness level. Figure 11 illustrates the procedure of the aPVT experimental paradigm adopted in this research. 

#### 2.6.3. Subjective Evaluation Scale (KSS)

Karolinska Sleepiness Scale (KSS) [46] (Table 4) is a valid scale for assessing subjective alertness responses to external environmental, circadian and pharmacological stimuli. In this research, the KSS was employed in conjunction with the EEG indicator *β* to evaluate alertness during various task phases. Notably, in the experiment, since the drivers were not excessively drowsy and could not realistically rate themselves as ‘completely unable to stay awake’, the 10th option, which is not part of the standard KSS, was omitted. Consequently, the KSS scores in this study range from 1 to 9 points. A higher KSS score corresponds to a lower level of individual alertness, signifying increased sleepiness.

## 3. Results

### 3.1. EEG (β)

The results of the three-factor repeated measures ANOVA showed that the main effects of CCT, Illuminance, and Task [F(2, 34) = 32.778, *p* < 0.001, η^2^ = 0.658; F(1, 17) = 6.387, *p* = 0.022, η^2^ = 0.273; F(2, 34) = 9.799, *p* < 0.001, η^2^ = 0.366, respectively] were significant.

**Figure 12:**
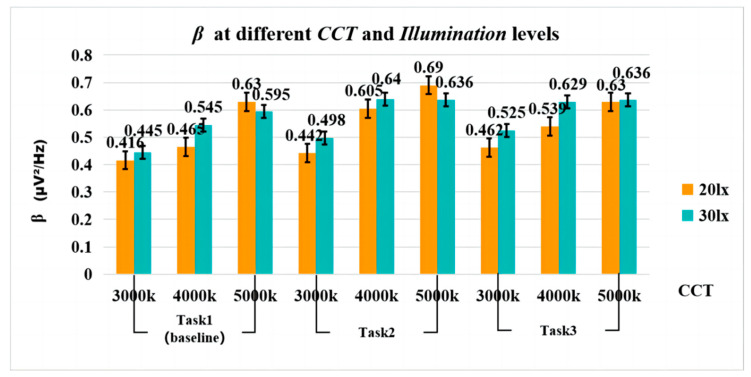
*β* at different *CCT* and *Illumination* levels.

The interaction effects of *CCT × Illuminance* was significant [F(2, 34) = 8.975, *p* = 0.001,η^2^ = 0.346] (see Figure 12). Post-hoc comparisons showed that with a fixed CCT of 3000K, *β* was significantly reduced for 20 lx (vs. 30 lx) (*p* = 0.002); Similarly, with a fixed CCT of 4000K, *β* was significantly reduced for 20 lx (vs. 30 lx) (*p* = 0.002). When the illuminance is fixed at 20 lx, *β* was significantly lower at 3000K (vs. 4000K; 5000K) (*p* = 0.001; *p* < 0.001) and 4000K compared to 5000K; *β* was significantly lower (*p* = 0.002). At a fixed illuminance of 30 lx, *β* was significantly lower at 3000K (vs.4000K; 5000K) (*p* < 0.001; *p* < 0.001).

The interaction effects of *CCT × Task* was significant [F(4, 68) = 2.781, *p* = 0.033, η^2^ = 0.141]. Post-hoc comparisons showed that in Task 1, *β* was significantly lower for 3000K (vs. 4000K; 5000K) (*p* < 0.001); 4000K vs. 5000K, *β* was significantly lower (*p* < 0.001). In Task 2, *β* was significantly lower at 3000K (vs. 4000K; 5000K) (*p* = 0.001; *p* < 0.001). During Task 3, *β* was significantly lower at 3000K (vs. 4000K; 5000K) (*p* = 0.011; *p* < 0.001).

The interaction effects of *Illuminance × Task* [F(2, 34) = 1.648, *p* = 0.207, η^2^ = 0.088] or *CCT × Illuminance × Task* [F(2.555, 43.429) = 0.479, *p* = 0.669, η^2^ = 0.027] was not significant (see Figure 12).

### 3.2. Reaction Time (RT)

**Figure 13:**
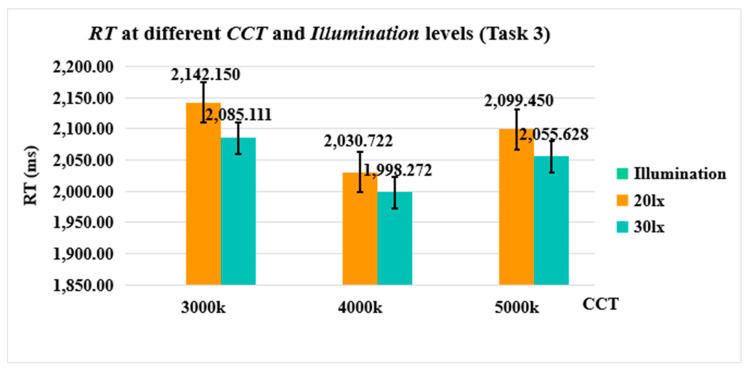
*RT* at different *CCT* and *Illumination* levels (Task3).

Since *RT* was only measured at Task 3, a two-way measurement ANOVA was employed, revealing that the main effects of *CCT*, *Illumination* and their interaction effects (*CCT × Illumination*) were not significant [F(2, 34) = 1.432, *p* = 0.253, η^2^ = 0.082; F(1, 17) = 1.141, *p* = 0.300, η^2^ = 0.063; F(2.912, 58.246) = 0.714, *p* = 0.543, η^2^ = 0.034, respectively] (see Figure 13).

### 3.3. Subjective Evaluation Results (KSS)

The results of the three-factor repeated measures ANOVA showed that the main effect of *CCT* [F(2, 34) = 4.414, *p* = 0.020, η^2^ = 0.206] was significant, while the main effect of *Illuminance* or *Task* [F(1, 17) = 4.402, *p* = 0.051, η^2^ = 0.206; F(2, 34) = 0.400, *p* = 0.674, η^2^ = 0.023, respectively] was not significant.

**Figure 14:**
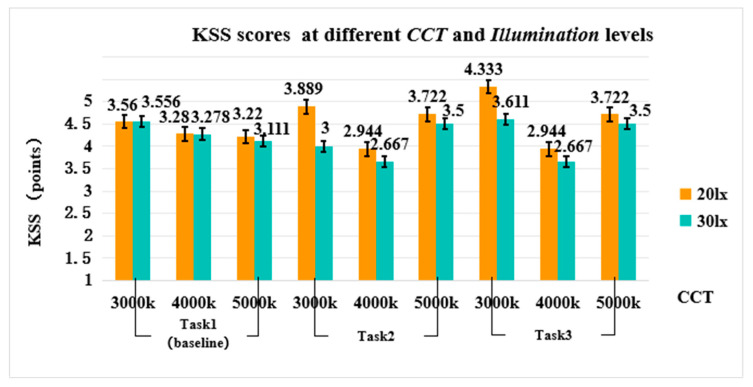
KSS scores at different *CCT* and *Illumination* levels.

The interaction effects of *CCT × Task* were significant [F(4, 68) = 0.357, *p* = 0.001, η^2^ = 0.242] (see Figure 14), and post-hoc comparisons showed that during the Task 2 phase, KSS scores were significantly higher in the 3000K (vs. 4000K) (*p* = 0.024); 4000K was significantly lower than 5000K (*p* = 0.04). During the Task 3 phase, KSS scores in the 3000K (vs. 4000K) were significantly higher (*p* = 0.001); and significantly lower in 4000K (vs. 5000K) (*p* = 0.003). Given that higher KSS scores reflect reduced levels of alertness, the aforementioned findings suggest that participants reported significantly higher subjective alertness for 4000K compared to the 3000K (during the Task 2 phase) and the 5000K (during the Task 3 phase).

The interaction effects of *CCT × Illuminance* [(F(2, 34) = 0.796, *p* = 0.459, η^2^ = 0.045), *Illuminance × Task* [F(2, 34) = 2.064, *p* = 0.143, η^2^ = 0.108], and *CCT × Illuminance × Task* [F(4, 68) = 1.108, *p* = 0.360, η^2^ = 0.061] were not significant (see Figure 14).

## 4. Discussion

The EEG evaluations revealed a significant interaction between *CCT* and *Illuminance*, as well as between *CCT* and *Task.* The results of the interaction of *CCT × Illuminance* showed that elevated illuminance levels (30 lx vs. 20 lx) significantly increased subjects’ alertness (*β*) at 3000K and 4000K. However, a parallel trend was not evident at 5000K. This discrepancy may be attributed to the fact that light sources with high CCT contain a higher component of short-wave blue light, which can drive ipRGCs with a comparable intensity to that of the illuminance (20 lx, 30 lx). Consequently, there was limited variation in the impact of fine-tuning illuminance (20 lx, 30 lx) on driver alertness under high CCT (5000K). Additionally, under low illuminance(20 lx), increasing CCT proved advantageous in enhancing driver alertness, with significant differences observed between 3000K vs. 4000K (*p* = 0.001), 3000K vs. 5000K (*p* < 0.001), and 4000K vs. 5000K (*p* = 0.002). Under high illuminance (30 lx), the medium CCT (4000K) also significantly increased driver alertness in comparison to the low CCT (3000K). It is noteworthy that there was no significant change in participants’ alertness when exposed to 4000K and 5000K, possibly due to the similar short-wave distribution characteristics of the 4000K and 5000K, which stimulated ipRGCs to a similar extent under high light conditions.

The results of the interaction between *CCT* and *Illuminance* showed that during Task 2 and Task 3, participants exhibited higher levels of alertness in 4000K and 5000K compared to 3000K. Figure 12 shows that participants’ alertness improved as CCT increased, aligning with prior research findings [20,47,48,49].

The subjective alertness ratings revealed that only the interaction between *CCT* and *Task* significantly impacted participants’ subjective alertness. Specifically, during Task 2 and Task 3, participants reported notably higher subjective alertness scores in both the 3000K and 5000K compared to the 4000K. This implies that maintaining a medium CCT (4000K) facilitated a more favorable subjective vigilance and attentive state during driving tasks involving frequent stops at red lights, traffic congestion, and car-following scenarios. A comprehensive comparative analysis of the EEG and subjective evaluations displayed a high level of alertness, demonstrating a general trend of heightened alertness among subjects with increasing CCT. However, no discernible modulation effect of CCT on subjective alertness was observed during the initial phase of the task. In this context, it is hypothesized in this paper that the impact of CCT on drivers’ subjective alertness might also be influenced by the duration of the light stimulus [17,50,51]. Characterized by limited-duration light exposure, no significant differences in subjective alertness among subjects in various CCTs were observed. To summarize, these findings align with prior studies concluding that appropriate increments in illuminance (30 lx) and CCT (4000K, 5000K) can enhance individual alertness [52,53]. Nonetheless, further exploration is required to determine whether additional increases in CCT or illuminance might disrupt drivers’ cognitive efficiency.

*RT* measurements showed that neither the interaction between *CCT* and *Illuminance* nor the main effect of *CCT* and *Illuminance* reached statistical significance. This suggests that drivers’ *RT* to random stimuli were not substantially influenced by external lighting conditions. Several factors may contribute to this observation. Firstly, it may relate to the specific nature of the experimental task. For instance, a study by Ru et al. (2019) noted significant non-visual effects of illumination on *RT* during Go/No-go and Flanker tasks, yet illumination did not notably affect *RT* during the PVT. Secondly, it might stem from the subjective attitudes of the participants towards the task. Participants might not have felt a significant sense of urgency when faced with sudden stimuli in the virtual driving scenario, resulting in minimal differences in their *RT* across various lighting conditions. Figure 14 reveals that, regardless of CCT variations, *RT* was higher under high illumination (30 lx) compared to low illumination (20 lx). This implies that elevating the illuminance of urban motor vehicle road lighting may not necessarily enhance drivers’ immediate responsiveness to unforeseen situations, aligning with findings from [53]. The mechanism behind this phenomenon remains unclear and warrants further investigation. In addition, as *CCT* increased, *RT* displayed a pattern of initially shortening and then lengthening. This trend suggests that a moderate CCT (around 4000K) might be most favorable for drivers to respond promptly when confronted with unexpected driving circumstances.

## 5. Conclusions

Previous studies have extensively explored and documented the immediate impacts of illuminance and CCT within indoor or tunnel interior lighting on individual alertness and task performance. However, limited research has delved into the repercussions of nighttime outdoor roadway lighting on non-visual effects on driver alertness. Moreover, the interactive effects of illuminance and CCT on individual cognitive function have rarely been discussed in previous studies. Therefore, this research focused on the mechanisms through which illuminance and CCT, along with their interactions, influence driver alertness in urban motor vehicle road lighting during nighttime (19:00~20:30). The main conclusions of this study are as follows:

(1) In summary, appropriately elevated illuminance (30 lx vs. 20 lx) and CCT (4000K and 5000K vs. 3000K) are more conducive to improving driver alertness and positively impacting driving task performance.
(2) EEG measurements revealed a significant impact of the interaction between CCT *×* Illuminance and CCT *×* Task on drivers’ alertness (*β*). Moreover, a significant interaction effect of CCT *×* Task on drivers’ subjective alertness was observed. However, no significant main effects of CCT and Illuminance on reaction time (*RT*) across driving tasks were identified. Nevertheless, observational data indicated that drivers exhibited the shortest reaction time under lighting conditions of 4000K CCT and 30 lx illuminance. This suggests that this specific combination of lighting may help reduce driver reaction time.

## Figures and Tables

**Table 1:** Research variables and parameterization.

	Variable	Level	Parameters
Independent variable	CCT	3	3000K, 4000K, 5000K
	Illumination (the average illuminance of the road surface)	2	20 lx, 30 lx
	Task	3	Task 1 (baseline): Monotonous driving. Task 2: Waiting for traffic lights and experiencing traffic congestion. Task 3: Auditory Psychomotor Vigilance Task (aPVT; Dual-task experimental paradigm)
Implicit variable	EEG (β waves)		
	Reaction time (RT)		

**Table 2:** The measurement results of illuminance on actual roads.

	Lane 1 L1	Lane 2 L2	Lane 3 L3	Lane 4 L4	The Average Illuminance (Eye-Level) of the Entire Road = (L1 + L2 + L3 + L4)/4
Dynamic average (eye-level) illuminance (lx)	3.857	2.771	2.706	2.942	3.069
Static average (eye-level) illuminance (lx)	3.214	3.229	3.100	3.229	3.193
Maintained average illuminance of road surface (lx)	28.5				

**Table 3:** Parameter settings for simulation of the virtual light source models.

Setting Objects	Parameters		
CCT (Actual Luminaires)	3000k	4000k	5000k
RGB (3D Max models)	(255, 161, 72)	(255, 209, 163)	(255, 228, 206)

**Table 4:** Karolinska Sleepiness Scale (KSS).

Item	Score
	1
Very alert	2
Alert	3
Rather alert	4
Neither alert nor sleepy	5
Some signs of sleepiness	6
Sleepy, but no effort to keep awake	7
Sleepy, but some effort to keep awake	8
Very sleepy, great effort to keep awake, fighting sleep	9
Extremely sleepy, can’t keep awake	10

## Data Availability

The data that support the findings of this study are available on request from the corresponding author.
